# A rare complication in percutaneous nephrolithotomy: clinical case and implications

**DOI:** 10.1093/jscr/rjae177

**Published:** 2024-03-21

**Authors:** Saleh Abdelkerim Nedjim, Hissein Hagguir Berdé, Adil Kbirou, Amine Moataz, Mohamed Dakir, Adil Debbagh, Rachid Aboutaieb

**Affiliations:** Urology Department, CHU Ibn Rochd, Casablanca 20100, Morocco; Urology Department, CHU Ibn Rochd, Casablanca 20100, Morocco; Urology Department, CHU Ibn Rochd, Casablanca 20100, Morocco; Urology Department, CHU Ibn Rochd, Casablanca 20100, Morocco; Urology Department, CHU Ibn Rochd, Casablanca 20100, Morocco; Urology Department, CHU Ibn Rochd, Casablanca 20100, Morocco; Urology Department, CHU Ibn Rochd, Casablanca 20100, Morocco

**Keywords:** kidney stone, percutaneous nephrolithotomy, complication, hemorrhage, lumbar artery

## Abstract

Percutaneous nephrolithotomy has become the standard procedure for the management of large kidney stones. Compared with other endo-urological techniques, it has a better fragmentation rate in a single session for kidney stones over 20 mm. It is therefore the recommended first-line treatment modality for large kidney stones. Bleeding is a well-known complication of this procedure, often requiring transfusion. In 0.8% of cases, bleeding can be severe, requiring surgical intervention to control hemostasis. Damage to the lumbar artery is a very rare event, and a potential complication. To our knowledge, this is the first report of lumbar artery involvement during percutaneous nephrolithotomy, complicated by severe bleeding and hemodynamic instability, necessitating conversion. Surgical exploration revealed a severed and bleeding lumbar artery. After hemostasis control by coagulation and ligation, the patient became stable. The patient was discharged on D3, where ureteroscopic lithotripsy was planned after collegial discussion with the patient. Based on this experience, it is important for the surgeon to have in mind certain principles and a thorough knowledge of the classic lateral lumbotomy approach to the kidney.

## Introduction

First described in 1976 by Fernström and Johansson [1], percutaneous nephrolithotomy (PCN) has now become the standard procedure for the management of large kidney stones [2]. Compared with other endo-urological techniques, it has a better fragmentation rate in a single session for kidney stones over 20 mm. It is therefore the recommended first-line treatment modality for large kidney stones [3, 4]. Although 1/5 of patients experience a complication [5], it is considered an effective and safe operation. And is rarely associated with severe bleeding secondary to vascular compromise [6]. As with percutaneous nephrostomy, PCNL-related hemorrhage is generally associated with injury to the renal artery and its branches, such as the segmental or interlobar arteries. Lesion of the lumbar artery is a very rare event [7]. In 0.8% of cases, surgical intervention (ranging from embolization to nephrectomy) is required to control bleeding in the event of severe hemorrhage [8]. In this article, we report a case of severe hemorrhage during the dilatation stage, requiring surgical exploration and revealing damage to the lumbar artery.

## Case history

**Figure 1:**
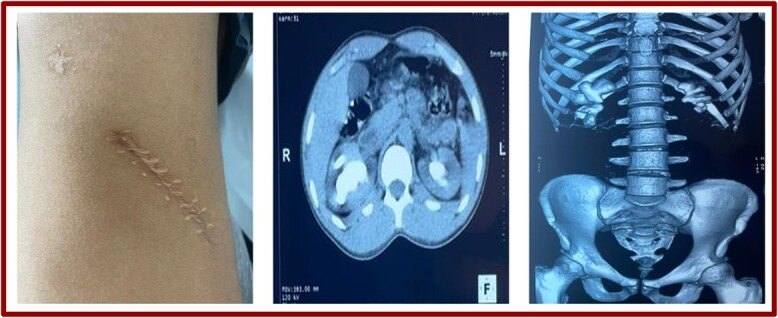
Lumbotomy scar and scans (coronal sections and reconstruction).

This 27-year-old patient, who had undergone lumbotomy surgery for renal lithiasis 10 years previously, presented to a urology consultation with bilateral low-back pain associated with emission of stone fragments during micturition, which had been evolving for 2 months. This low-back pain was not associated with fever or other lower urinary tract signs. Examination revealed a patient in very good general condition, with a body mass index of 20. A right lumbotomy scar was present (Fig. 1), and there was mild lumbar pain on palpation and percussion.

Biologically, hemoglobin was 12 mg/l and plasma creatinine 17 g/dl. Hemostasis was unremarkable. An injection-free abdominopelvic CT scan revealed normal-sized, regularly contoured, well-differentiated kidneys, secreting and excreting within normal timeframes. Coralliform calculi were found in two kidneys measuring 6 cm on the right and 4 cm on the left, with a density of 631 HU on the right and 728 HU on the left (Fig. 1).

It was decided at a multidisciplinary urinary calculus meeting to perform a right PCNL. The patient was taken to the OR after an anesthetic evaluation and sterile urinalysis.

After general anesthesia, orotracheal intubation, and injection of tranexamic acid, the patient was positioned as described by Lezrek *et al.* [9]. After cystoscopy (normal: no calculus), a ureteral catheter was inserted, followed by bladder catheterization. Puncture of the inferior calyx was performed under ultrasound guidance. The methylene blue injected by the ureteral probe exited through the puncture needle. A guide wire was inserted into the needle and then removed. Progressive dilatation with progressive-gauge dilators (presence of blood), followed by an Amplatz dilator, then placement of the access sheath. After removal of the Amplatz dilator, significant bleeding was observed, with warm, bright-red, pulsatile blood. Exploration of the calyces with the nephroscope was hampered by the bleeding, despite extensive afloat irrigation and injection of tranexamic acid. It was decided to remove the access sheath, leaving a guide in place. Bleeding decreased after 5 min of lumbar fossa compression.

Resumption of the procedure after dilatation on the guide already in place and placement of the access sheath. On reintroduction of the nephroscope, the stone was found at the inferior calcific level, with persistent heavy bleeding. The patient began to desaturate, with severe hypotension (70/40 mm Hg) and tachycardia requiring noradrenaline administration. Capillary hemoglobin was 7 g/dl.

**Figure 2:**
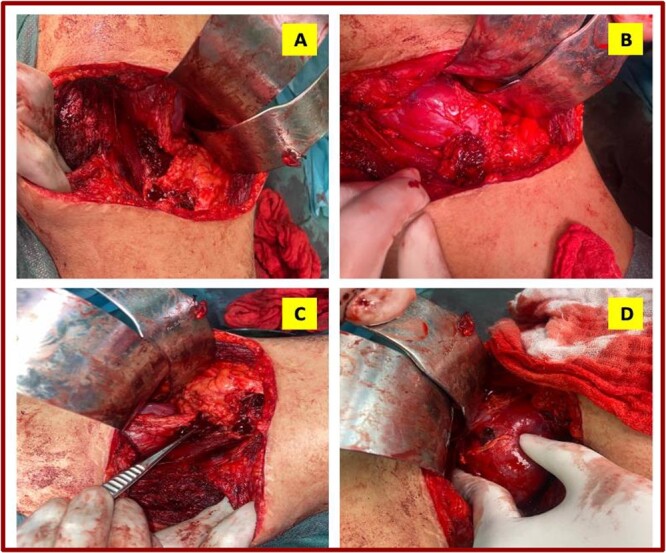
Intraoperative images showing exploratory finding.

Conversion to surgery was decided after bleeding persisted despite removal of the hardware, compression of the lumbar region and instability despite drugs. In the initial position, an incision was made over the old lumbotomy scar. Fibrosis of the lumbar wall was noted, with infiltration by irrigation fluid. After pushing back the peritoneum and exposing the retroperitoneum, there was a large retroperitoneal hematoma and the psoas (Fig. 2A) was respected. There was also a significant hematoma of the peri-renal fat (Fig. 2B). After freeing the posterior aspect of the kidney with the lumbar wall, a severed and bleeding lumbar artery was identified (Fig. 2C). It was coagulated and ligated with 2/0 vircyl wire crosswise (X). Release of the renal fat to the inner edge of the kidney revealed the renal vessels, which were unharmed. The kidney showed good staining, with evidence of the puncture site and dilatation (Fig. 2D).

With norepinephrine, transfusion of two packed red blood cells and control of hemostasis, the patient was stabilized. A redon drain was inserted. Extraction of the calculus was not performed, as the cast of the calculus necessitated nephrotomy of a previously operated kidney. Still in the same position, a double-J catheter was inserted after removal of the ureteral catheter. At the end of the operation, the patient was transferred to intensive care with noradrenaline. At D1, the patient was conscious, hemodynamically and respiratorily stable. The dressing was clean and the abdomen supple. Diuresis was 2 l. The redon drain brought back 30 cc of blood. Hemoglobin was 9 mg/l and creatinine 14 g/dl. At D2, the redon had returned ~30 cm. The bladder catheter was removed. On D3 post-op, the patient was discharged from hospital. After discussion with the patient and as a team, it was decided to plan a multi-session laser ureteroscopic lithotripsy for stone fragmentation.

## Discussion

With a success rate of over 90%, PCN has an overall complication rate of up to 83%. Complications include bleeding, requiring transfusion in 11.2 to 17.5% of cases [10]. In 0.8% of cases, bleeding can be severe, requiring surgical intervention to control hemostasis [8]. In this report, we present a case of bleeding complicated by hemodynamic instability, requiring conversion for surgical exploration.

Bleeding during and after PCNL can occur at any stage of the procedure: puncture, percutaneous dilatation, dilatation and introduction of the access sheath, aggressive stone handling and extraction, removal of the nephrostomy tube, and in the immediate or late postoperative period [8]. Bleeding is rarely due to damage to the main vessels of the kidney, but is more often secondary to damage to segmental arteries or small intrarenal vessels [11, 12]. In most cases, bleeding is venous in origin [13]. Involvement of the lumbar artery is rare [7], and is a potential complication as described in trans psoas spinal surgery [14]. The characteristics of the bleeding reported in our case immediately suggest arterial involvement.

On 11 November 2023, by entering the terms “Bleeding, lumbar artery and percutaneous nephrolithotomy” on Pubmed, 121 results were available, including a single article reporting 2 cases of bleeding due to lumbar artery involvement in connection with percutaneous nephrolithotomy. The bleeding occurred on postoperative day 6, when the percutaneous nephrostomy was removed [15]. In our case, the bleeding occurred during the dilatation procedure.

Several parameters have been identified as risk factors for bleeding during or after PCNL. These include: age, gender, body mass index, presence of comorbidities such as hypertension, diabetes mellitus, serum creatinine level, history of renal surgery and type of stone, stone burden, extent of hydronephrosis and surgeon experience [16]. Other parameters have also been reported: ASA grade, duration of operation and multiple punctures [17]. More than three factors are present in the present case.

It has been reported by Batagello *et al.* [18] that a single dose of tranexamic acid at the time of anesthetic induction is safe and reduces the need for blood transfusion by a factor of five, and can be used as standard clinical practice for patients with complex kidney stones undergoing PCNL. To manage intraoperative hemorrhage from PCN, a variety of techniques have been described in the literature. Strategies range from conservative stance, embolization, or open surgery [19].

In the event of hemodynamic instability and difficulty in visualizing the stone intraoperatively, a clamped nephrostomy tube can be inserted and the operation postponed. With a clamped nephrostomy, a clot will form, requiring compression by tamponade to stop bleeding. Strict monitoring of vital parameters is essential. Hemodynamic instability after resuscitation requires either urgent conventional renal angiography with super-selective embolization of the hemorrhagic vessel, or urgent open surgical exploration if angiography is not available [17]. Intraoperatively, persistent hemodynamic instability despite attempts at resuscitation and hemostasis should call for an aggressive surgical attitude to control hemostasis. Exploration of the renal pedicle must be carried out delicately and as quickly as possible.

## Conclusion

This incident highlights a crucial lesson for surgeons in the early stages of their experience: the need for in-depth knowledge of the techniques and risks associated with every surgical procedure. In this specific case, involvement of the lumbar artery during PCN resulted in a critical situation requiring surgical conversion and immediate intervention to control the hemorrhage and stabilize the patient. This rare complication underlines the importance of surgeons remaining vigilant and prepared for unforeseen situations, even during routine procedures. By having a comprehensive knowledge of anatomy and approach techniques, as well as an understanding of potential complications, surgeons can better anticipate and manage emergency situations, ensuring optimal outcomes for their patients.
